# A Soluble Factor from *Trypanosoma cruzi* Inhibits Transforming Growth Factor-ß-Induced MAP Kinase Activation and Gene Expression in Dermal Fibroblasts

**DOI:** 10.1371/journal.pone.0023482

**Published:** 2011-09-08

**Authors:** G. Adam Mott, Jaime A. Costales, Barbara A. Burleigh

**Affiliations:** Department of Immunology and Infectious Diseases, Harvard School of Public Health, Boston, Massachusetts, United States of America; The University of Maryland, United States of America

## Abstract

The protozoan parasite *Trypanosoma cruzi,* which causes human Chagas' disease, exerts a variety of effects on host extracellular matrix (ECM) including proteolytic degradation of collagens and dampening of ECM gene expression. Exposure of primary human dermal fibroblasts to live infective *T. cruzi* trypomastigotes or their shed/secreted products results in a rapid down-regulation of the fibrogenic genes collagenIα1, fibronectin and connective tissue growth factor (CTGF/CCN2). Here we demonstrate the ability of a secreted/released *T. cruzi* factor to antagonize *ctgf/ccn2* expression in dermal fibroblasts in response to TGF-ß, lysophosphatidic acid or serum, where agonist-induced phosphorylation of the mitogen-activated protein (MAP) kinases Erk1/2, p38 and JNK was also inhibited. Global analysis of gene expression in dermal fibroblasts identified a discrete subset of TGF-ß-inducible genes involved in cell proliferation, wound repair, and immune regulation that are inhibited by *T. cruzi* secreted/released factors, where the genes exhibiting the highest sensitivity to *T. cruzi* are known to be regulated by MAP kinase-activated transcription factors. Consistent with this observation, the Ets-family transcription factor binding site in the proximal promoter region of the *ctgf/ccn2* gene (−91 bp to −84 bp) was shown to be required for *T. cruzi*-mediated down-regulation of *ctgf/ccn2* reporter expression. The cumulative data suggest a model in which *T. cruzi*-derived molecules secreted/released early in the infective process dampen MAP kinase signaling and the activation of transcription factors that regulate expression of fibroblast genes involved in wound repair and tissue remodelling, including *ctgf/ccn2*. These findings have broader implications for local modulation of ECM synthesis/remodelling by *T. cruzi* during the early establishment of infection in the mammalian host and highlight the potential for pathogen-derived molecules to be exploited as tools to modulate the fibrogenic response.

## Introduction

The kinetoplastid protozoan parasite *Trypanosoma cruzi* causes Chagas' disease in humans, a chronic and debilitating condition affecting several million individuals in Latin America. *T. cruzi* is transmitted by an insect vector which gains access to the host via breaches in the skin or through mucosal membranes, mainly conjunctival or gastric mucosa [1], [2]. As an obligate intracellular parasite that disseminates from initial infection sites to tissues such as heart and smooth muscle, *T. cruzi* undergoes multiple rounds of invasion, growth and egress from infected cells during the acute stage of infection. Very little is currently known regarding the early interactions between *T. cruzi* and its host that facilitate establishment of infection *in vivo*. Cellular models of *T. cruzi* infection have been very useful for defining the molecular and cellular events that regulate the early parasite-host cell interactions and host cell invasion. During its early interaction with mammalian host cells, trypomastigotes, the invasive forms of *T. cruzi,* trigger rapid changes in a number of cellular signaling pathways to facilitate the process of parasite entry into non-professional phagocytic cells (reviewed in [3], [4]). While these early signaling events have been relatively well-studied in the context of *T. cruzi* invasion, little is known regarding the impact of these parasite-induced signaling cascades downstream of the invasion process. Transcriptional profiling of *T. cruzi*-infected fibroblasts revealed that the earliest detectable changes triggered by infective *T. cruzi* trypomastigotes involve down-regulation of a small subset of genes including members of the CCN family (*cyr61* and *ctgf/ccn2*) [5], which play important roles in angiogenesis and extracellular matrix (ECM) homeostasis [6]. *T. cruzi*-dependent dampening of *ctgf/ccn2* expression occurs at both the mRNA and protein levels and is mediated by a secreted/released parasite factor that is capable of antagonizing TGF-ß-mediated induction of *ctgf/ccn2* [7].

Connective tissue growth factor (CTGF/CCN2) is a 38 kDa secreted cysteine-rich heparin-binding glycoprotein [8] that promotes cell proliferation and co-operates with TGF-ß to promote myofibroblast differentiation and enhanced extracellular matrix (ECM) synthesis (reviewed in [9]). Dysregulation of CTGF/CCN2 expression leads to excessive scarring and fibrosis and this cytokine is over-expressed in a variety of tumors where CTGF/CCN2 levels correlate with disease progression [9]. As such there has been significant interest in CTGF/CCN2 as a therapeutic target for a number of disease states [10], [11], [12], [13]. Our finding that the human pathogen, *Trypanosoma cruzi,* releases a factor that inhibits TGF-ß-mediated expression of CTGF/CCN2 prompted further investigation into the mechanistic basis for this observation. CTGF/CCN2 expression is induced by diverse extracellular stimuli, including growth factors, cytokines and mechanical stress [9], [14], [15], [16], [17]. TGF-ß-stimulated expression of CTGF/CCN2 requires the activation of SMAD proteins and MAP kinases downstream of the TGF-ß receptor [9], [15]. Erk1/2 and p38 are generally associated with positive regulation of CTGF/CCN2 expression in different cell types [15], [18], [19], [20], whereas the role of JNK is more variable [15], [18], [20]. The expression of CTGF/CCN2 is also controlled via the activities of the ETS family of transcription factors. A functional Ets-binding site identified in the proximal *ctgf/ccn2* promoter, spanning the region −91 to −84 bp upstream of the transcriptional start site, is bound by Ets-1 to promote TGF-ß-dependent induction of *ctgf/ccn2* [21], while binding of the same site by Fli-1 negatively regulates *ctgf/ccn2* expression in human fibroblasts [22]. The activity of many members of the ETS family is controlled through MAP kinase signaling, where the DNA-binding and trans-activation activities of ETS transcription factors are regulated by phosphorylation [23], [24].

In the present study, we demonstrate that *T. cruzi*-dependent abrogation of *ctgf/ccn2* expression in human dermal fibroblasts is associated with inhibition of both basal and agonist-induced activation of MAP kinase signaling and requires the functional Ets-binding site in the proximal promoter of the *ctgf/ccn2* gene. Expanding our analysis of the impact of *T. cruzi* released factors on TGF-ß-induced fibroblast gene expression we describe a discrete subset of agonist-inducible fibroblast genes that are sensitive to *T. cruzi* secreted/released factors. We report that the group of TGF-ß-inducible genes that exhibit the highest sensitivity to a *T. cruzi* secreted/released fraction are MAP kinase-regulated genes that function in wound repair, extracellular matrix remodelling and host response pathways. Collectively, these findings provide novel insights into early *T. cruzi*-host cell interactions. We demonstrate the ability of secreted/released parasite molecules to hinder agonist-induced host cell signaling pathways and gene expression. If similar events occur early in the *T. cruzi* infection process *in vivo*, it would be predicted that local inhibition of ECM synthesis would facilitate dissemination from initial sites of infection.

## Results

### A secreted/released trypanosome factor inhibits agonist-induced expression of CTGF/CCN2 in human dermal fibroblasts

**Figure 1:**
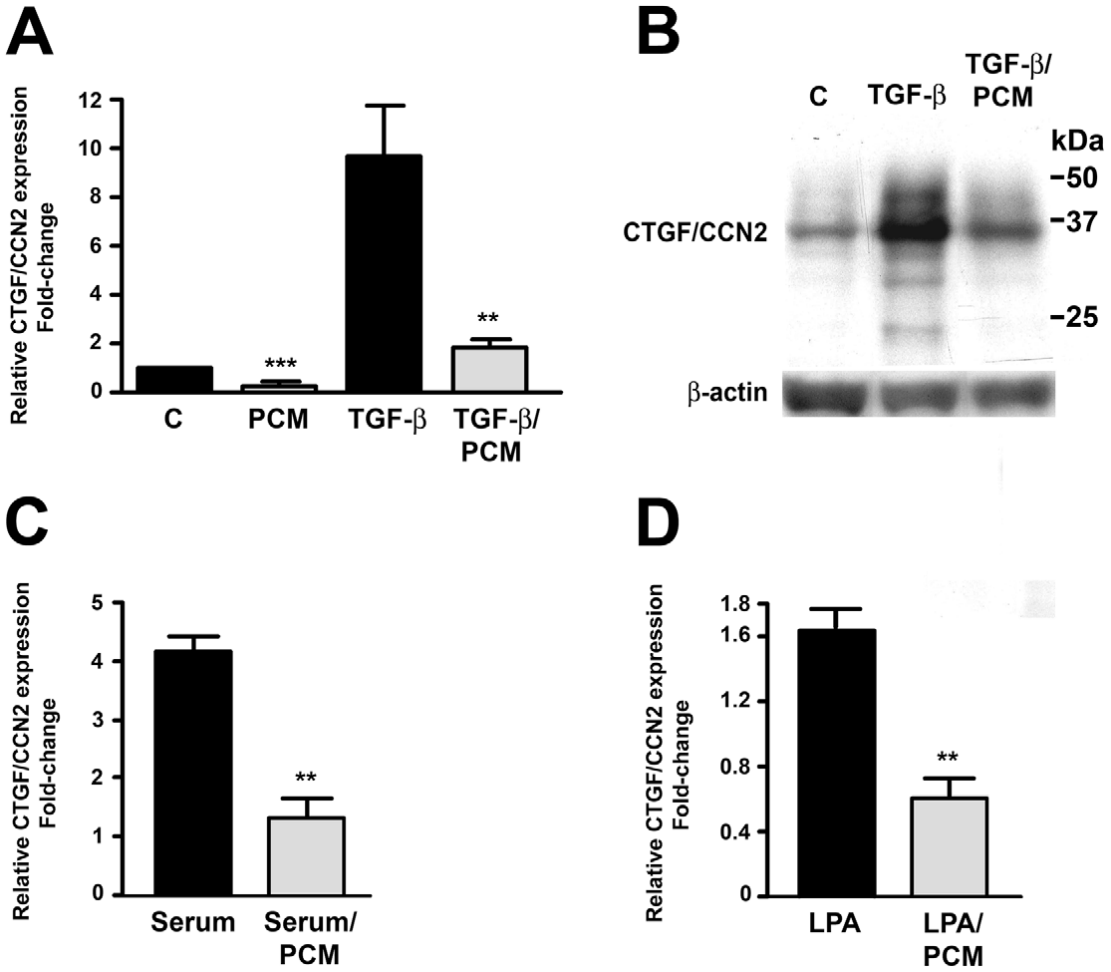
*T. cruzi* PCM inhibits agonist-induced ctgf/ccn2 expression. HFF were incubated with medium or *T. cruzi* PCM, alone or in combination with 5 ng/ml TGF-ß, 2 hours prior to mRNA harvest for quantitative real-time PCR analysis (A) or 24 hours prior to analysis of CTGF/CCN2 protein levels by western blot (B). *ctgf/ccn2* mRNA levels were analyzed by quantitative real-time PCR following stimulation of HFF with serum (2% v/v) (C) or 10 µM LPA (D) for 2 hours in the presence or absence of *T. cruzi* PCM. Data is represented as the mean ± S.E. from 5 independent experiments carried out in duplicate. Statistical significance was assessed using the Student's t-test, (** p<0.01).

Connective tissue growth factor (CTGF/CCN2) expression is rapidly repressed in human dermal fibroblasts infected with the protozoan parasite, *Trypanosoma cruzi,* where the CTGF/CCN2-repressive activity was shown to be associated with a secreted/released trypanosome factor that is present in parasite-conditioned medium (PCM) [5] [7]. While the repressive factor released from live infective *T. cruzi* trypomastigotes into the medium (parasite-conditioned medium; PCM) [7] has not been identified, it is associated with a trypsin-sensitive, heat-labile, high molecular weight protein fraction of *T. cruzi* PCM enriched in GPI-anchored surface proteins (A. Mott and B. Burleigh, unpublished data). Here we show that treatment of human dermal fibroblasts (HFF) with *T. cruzi* PCM blocks induction of *ctgf/ccn2* expression triggered by different agonists TGF-ß (Figure 1A, B), serum (Figure 1C) or lysophosphatidic acid (LPA) (Figure 1D). Thus, the ability of *T. cruzi* PCM to inhibit *ctgf/ccn2* expression in response to distinct exogenous agonists suggests that signaling molecules primarily associated with TGF-ß receptor signaling such as the SMADs, are unlikely to be the downstream target of *T. cruzi* PCM leading to the repression of *ctgf/ccn2*.

### *T. cruzi* PCM inhibits agonist-induced MAP kinase activation

**Figure 2:**
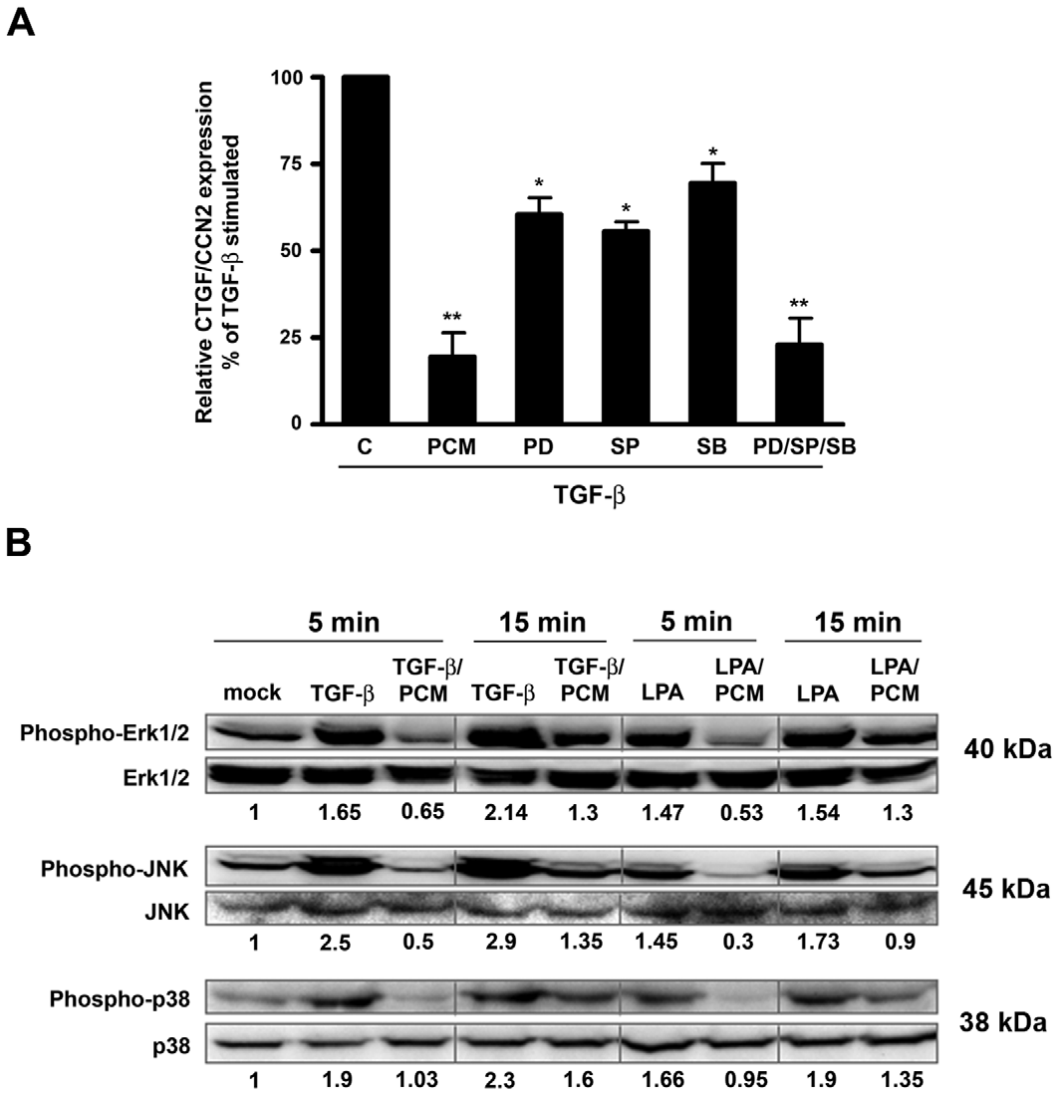
*T. cruzi* PCM abrogates MAP kinase activation and decreased MAP kinase signaling results in inhibition of ctgf/ccn2 expression. (A) HFF were stimulated with 5 ng/ml TGF-ß1 in the presence of medium, *T. cruzi* PCM or with 10 µM SP600125, 20 µM SB203580 or 50 µM PD98059 as indicated for 2 hours prior to mRNA harvest for quantitative real-time PCR analysis. MAP kinase inhibitor treatments were carried out following a 30-minute pre-incubation step. Data is represented as the mean ± s.e. from triplicate experiments (n = 4). Statistical significance was assessed using the Student's t-test (** p<0.01, * p<0.05). (B) Western blot of phospho-Erk, phospho-JNK and phospho-p38 normalized to total Erk, JNK and p38 respectively in lysates from HFF stimulated with 5 ng/ml TGF-ß1 or 10 µM LPA for 5 or 15 minutes in serum-free media or parasite-conditioned medium. Results for densitometric analysis are shown as numerical values below each panel and represented as phosphorylation relative to mock-treated controls (arbitrarily set to a value of 1.0).

Given the well-established role of MAP kinase signaling in the regulation of *ctgf/ccn2* expression in a variety of cell types [15], [18], [19], [20], [25], [26], Erk1/2, JNK and p38, were targeted in HFF with selective inhibitors to determine the impact on TGF-ß-inducible expression of *ctgf/ccn2.* Added individually, inhibitors of Erk1/2, JNK and p38 partially inhibited TGF-ß-dependent up-regulation of *ctgf/ccn2* whereas in combination, the inhibitors were much more effective (Figure 2A) suggesting that different arms of the MAP kinase signaling pathway are involved in regulation of *ctgf/ccn2* expression in HFF. As predicted from this result, stimulation of HFF with TGF-ß (5 ng/ml) or LPA (10 µM) promotes increased phosphorylation of Erk1/2, JNK and p38, which migrate on western blots at the expected molecular weights of 40 kDa, 45 kDa and 39 kDa respectively, where *T. cruzi* PCM abrogates both TGF-ß and LPA-induced phosphorylation of these kinases (Figure 2B). Combined, the data suggest that *T. cruzi* PCM exerts an inhibitory effect on *ctgf/ccn2* expression by dampening MAP kinase signaling pathways.

### An Ets binding site in the proximal *ctgf/ccn2* promoter is involved in *T. cruzi*-mediated repression of gene expression

**Figure 3:**
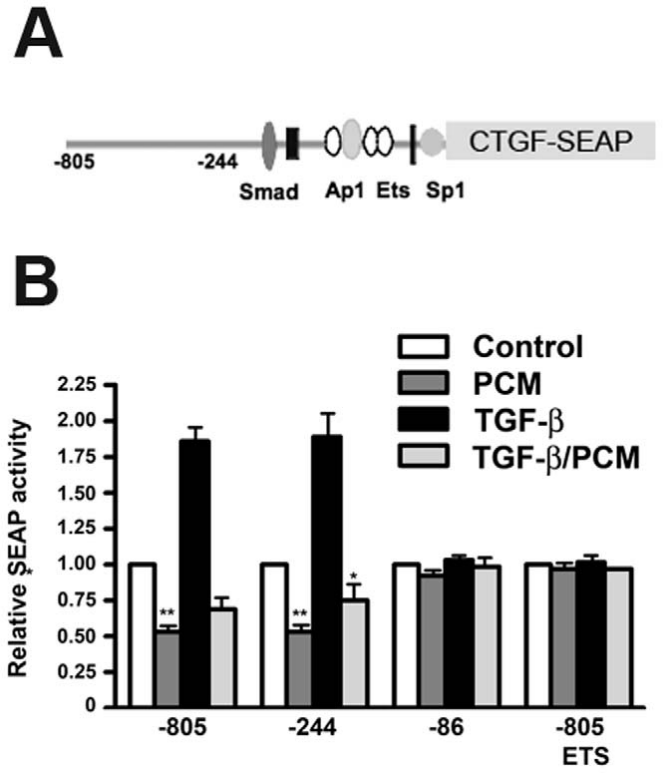
The Ets transcription factor binding site in the ctgf/ccn2 promoter is required for *T. cruzi* PCM-mediated repression of reporter expression. SEAP reporter constructs driven by nucleotides −805 to +17 (−*805*), −244 to +17 (−*244*), −86 to +17 (−*86*) or −805 to +17 with a mutated ETS sequence (−*805 ETS*) representing the proximal *ctgf/ccn2* promoter region (A) along with a ß-galactosidase containing control plasmid were transfected into HFF. (B) Serum-starved transfected cells were stimulated with medium alone or 5 ng/ml TGF-ß1 in the presence or absence of *T. cruzi* PCM for 6 hours. Relative reporter expression in treated cells relative to mock-treated controls is shown as the mean ± s.e. (n = 3). (*p<0.05, p<0.01).

The *ctgf/ccn2* promoter/enhancer region contains several protein-binding sites that have been implicated in regulation of gene expression [21], [22], [27], [28], [29], [30]. Focusing on the proximal promoter region of *ctgf/ccn2* (Figure 3A), we tested the impact of *T. cruzi* PCM treatment on basal and TGF-ß stimulated reporter activity using a series of *ctgf/ccn2* promoter/SEAP reporter plasmids [21], [27] transfected into human dermal fibroblasts. As previously described, reporters containing functional Smad3 and Ets-binding sites: *ie*. spanning regions -805 to +17 bp (Figure 3B; *−805*) or −244 to +17 bp (Figure 3B ; *−244*) of the *ctgf/ccn2* promoter were activated by TGF-ß whereas a severely truncated promoter/reporter spanning −86 to +17 bp of the *ctgf/ccn2* promoter (Figure 3B; *−86*) failed to activate following TGF-ß stimulation. *T. cruzi* PCM treatment resulted in a significant decrease in basal activity of the *−805* and −*244* reporter constructs and blocked TGF-ß mediated activation of these reporters (Figure 3B). In contrast, basal expression of the reporter from the truncated construct (Figure 3B; *−86*) did not change in response to *T. cruzi* PCM. These data suggest that the Ets site present at −91 to −84 bp upstream of the transcriptional start site in the promoter is required to mediate the repressive effects of *T. cruzi* PCM on *ctgf/ccn2* gene expression. In order to confirm this critical role for the Ets consensus site in PCM-mediated repression of *ctgf/ccn2* expression, we examined the effect of *T. cruzi* PCM on the full-length reporter containing a mutated Ets site (Figure 3B; *−805 ETS*). As previously reported, a functional Ets binding site is required for TGF-ß-stimulated *ctgf/ccn2* gene expression, and therefore this reporter is refractory to TGF-ß treatment (Figure 3B). Critically, a comparison between the response of the *−805* and the mutant *−805 ETS* to PCM demonstrates the inability of PCM to repress activity in the absence of a functional Ets binding site (Figure 3B). We conclude from these experiments that a functional Ets binding site in the *ctgf/ccn2* promoter is essential for TGF-ß dependent gene expression, and imparts sensitivity to *T. cruzi*-mediated inhibition of *ctgf/ccn2* gene expression.

### *T. cruzi* PCM inhibits the expression of a subset of MAP kinase-regulated fibroblast genes involved in wound repair

Results presented above demonstrate that the factor(s) that are secreted or shed by *T. cruzi* trypomastigotes antagonize MAP kinase signaling in HFF downstream of TGF-ß and LPA. To determine the global impact of *T. cruzi* PCM on both basal and TGF-ß stimulated gene expression in human dermal fibroblasts, transcriptomic analyses were conducted following exposure of HFF to TGF-ß (5 ng/ml) in the presence or absence of *T. cruzi* PCM for 3 hours. cDNA prepared from treated and control cells were hybridized to human Affymetrix arrays containing ∼47,000 probesets (HG_U_133, 2.0). Raw expression data from triplicate experiments was imported into, and processed using, Rosetta Resolver 7.0 as outlined in the Experimental Procedures. Genes with expression levels that differed by 2-fold or greater (p<0.01) between the different experimental groups were considered for further analysis.

Examining first the impact of the secreted/released *T. cruzi* fraction on fibroblast gene expression, we find 89 unique genes (in 121 probe-sets) consistently down-regulated in PCM-treated cells and 82 genes (in 107 probe-sets) up-regulated greater than 2-fold (Table S1). Many of the repressed genes are ‘immediate early’ genes that regulate transcription (*egr-1, jun, myc, fos, KLF2, KLF6*) and are involved in cell proliferation [31], [32], [33], [34], [35] and inflammation [36], [37], [38]. As predicted from our studies with *ctgf/ccn2, T. cruzi* PCM treatment also results in the down-regulation of genes that are involved in extracellular matrix synthesis and tissue remodeling including hyaluronan synthase 2 (*HAS2*), tenascin C (*TNC*) and the CCN family members *ctgf/ccn2* and *cyr61* [17].

**Figure 4:**
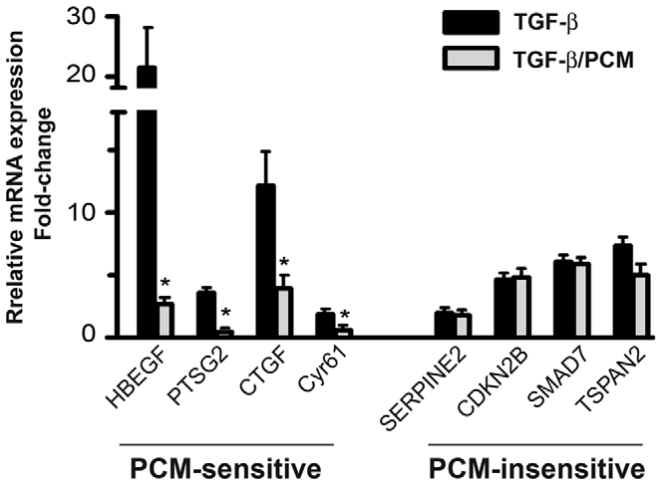
*T. cruzi* PCM inhibits the expression of a broader range of TGF-ß-inducible genes. Quantitative real-time PCR analysis of the relative expression of representative PCM-sensitive and PCM-insensitive TGF-ß-inducible genes in HFF after 2 hours of treatment with 5 ng/ml TGF-ß in media or PCM. Genes analyzed: HBEGF, heparin-binding EGF-like growth factor; PTSG2, prostaglandin-endoperoxide synthase 2; CTGF, connective tissue growth factor; Cyr61, cysteine-rich angiogenic factor; SERPINE2, plasminogen activator inhibitor type 1, member 2; CDKN2B; cyclin-dependent kinase 4 inhibitor B; SMAD7, MAD, mothers against decapentaplegic homolog 7; TSPAN2, tetraspanin 2.

*T. cruzi* PCM inhibited the expression of approximately 30% of the with TGF-ß-inducible genes in HFF (Table S2). The 59 genes appearing in Table 1 represent the annotated, non-redundant TGF-ß inducible genes for which expression was inhibited by 1.7-fold or greater by *T. cruzi* PCM. Among the most highly PCM-sensitive, TGF-ß inducible genes are those with known involvement in extracellular matrix remodeling, wound healing and angiogenesis including heparin-binding EGF-like growth factor (*HBEGF*), angiopoietin-like 4 (*ANGPTL4*), endothelin-1 (*EDN1*), tropomyosin 1 (*TPM1*), chondroitin 4-sulfotransferase (*CHST11*), early growth response 1 (*egr-1*), connective tissue growth factor (*ctgf/ccn2*) and cysteine-rich, angiogenic inducer, 61 (*CYR61*). In addition, interleukin 6 (*IL-6*), chemokine (C-C motif) ligand 2 (*CCL2*) and prostaglandin-endoperoxide synthase 2 (*PTGS2*), which have been grouped with PCM-sensitive genes involved in inflammation and immune regulation (Table 1), also play an important role in wound repair [39], [40], [41], [42]. In support of the proposed role for MAP kinase-inhibition in mediating the effects of *T. cruzi* PCM on fibroblast gene expression, the TGF-ß-inducible genes with the highest sensitivity to *T. cruzi* PCM-mediated repression in the microarray analysis (ie. those for which a 4-fold or greater inhibition of TGF-ß-mediated induction of target gene expression was observed: *EGR1* (17-fold), *HBEGF* (13-fold), *PTGS2* (8.2-fold), *LIF* (7-fold), *EDN1* (5.7-fold), *IL6* (5.5-fold), *CYR61* (4-fold)) are known to be regulated by MAP kinase activated transcription factors such as ETS-family transcription factors, AP-1 and NF-κB in a variety of cell types [43], [44], [45], [46], [47], [48], [49]. Quantitative RT-PCR carried out to confirm the pattern of PCM sensitivity for several TGF-ß inducible genes (Figure 4) reveals consistency with the microarray data where *T. cruzi* PCM was shown to inhibit TGF-ß dependent induction of *HBEGF, CTGF/CCN2, PTSG2* and *Cyr61*. In contrast, TGF-ß stimulated expression of *SERPINE2, CDKN2B, SMAD7* and *TSPAN2* were refractory to PCM treatment (Figure 4). These data highlight both the broader effect of *T. cruzi*-derived factors on fibroblast gene expression and reveal selectivity of the response as only a subset of TGF-ß-inducible genes were affected when cells were co-stimulated with *T. cruzi* PCM.

**Table 1:** *T. cruzi* PCM inhibits expression of a subset of TGFß-inducible genes.

Accession	Gene Symbol	Gene Description	*TGF-ß	TGF-ß +
				PCM
**ECM Remodeling/Angiogenesis**				
M60278	HBEGF	Heparin-binding EGF-like growth factor	50.4	3.7
AF169312	ANGPTL4	Angiopoietin-like 4	42.0	19.9
J05008	EDN1	Endothelin-1	7.3	1.3
BC020765	SERPINE1	PAI-1	6.7	3.9
AI459194	EGR1	Early growth response 1	5.3	0.3
M92934	CTGF	Connective tissue growth factor	4.2	2.4
NM_007036	ESM1	Endothelial cell-specific molecule 1	3.4	0.9
AI806905	CHST11	Chondroitin 4 sulfotransferase 11	2.9	1.7
AI524125	PCDH9	Protocadherin 9	2.7	1.6
BC036577	DDAH1	Dimethylarginine dimethylaminohydrolase 1	2.7	1.1
AI694545	PLXNA2	Plexin A2	2.6	1.1
AI860360	TPM1	Tropomyosin 1a	2.5	1.3
AI374739	HAS2	Hyaluronan synthase 2	2.3	1.2
Y13786	ADAM19	ADAM metallopeptidase domain 19	2.2	1.3
AB002373	RUSC2	RUN and SH3 domain containing 2	2.1	1.2
AF003114	CYR61	Cysteine-rich, angiogenic inducer, 61	2.1	0.5
**Inflammation/Immunity**				
NM_000600	IL6	IL-6	4.2	0.8
NM_000963	PTGS2	Prostaglandin-endoperoxide synthase 2	3.1	0.4
S69738	CCL2	Chemokine (C-C motif) ligand 2	2.6	0.9
AI608902	CD274	CD274 antigen	2.5	0.9
U93091	TLR4	Toll-like receptor 4	2.1	0.9
**Cell growth/differentiation**				
BC093735	LIF	Leukemia inhibitory factor	6.4	1.0
AK000850	NEDD9	Crk-associated substrate related	6.1	3.4
NM_030751	SNF1LK	SNF1-like kinase	3.7	2.1
H94882	ANKRD15	Ankryn repeat domain 15	3.7	1.8
M13436	INHBA	Inhibin, beta A	3.3	1.0
AK025317	MAML2	Mastermind-like 2	3.1	1.3
AB011109.1	NUAK1	NUAK family, SNF1-like kinase	3.0	1.4
NM_005118	TNFSF15	Tumor necrosis factor superfamily 15	2.7	1.3
AF329092	DOC1	Anaphase-promoting complex 1	2.4	0.8
AL136919	LRRC8C	Leucine rich repeat protein	2.3	1.3
**Signaling**				
AV686810	RHOB	RhoB	10.0	4.6
AF087853	GADD45B	Growth arrest and DNA-damage-inducible	5.5	2.5
BE302191	STK38L	Serine/threonine kinase 38 like	3.3	2.0
AU144916	IGFBP7	Insulin-like growth factor binding protein 7	3.2	1.3
NM_007368	RASA3	RAS p21 protein activator	2.8	1.7
NM_006823	PKIA	Protein kinase cAMP-dependent, catalytic) inhbitor	2.7	1.1
AI139993	SH3GLP3	SH3-domain GRB2-like	2.3	0.9
AU157259	SMURF2	SMAD specific E3 ubiquitin protein ligase 2	2.2	1.1
AB043703	FZD8	Frizzled homolog 8	2.2	0.8
NM_004073	PLK3	Cytokine-inducible kinase	2.2	0.8
**Transcriptional regulation**				
NM_000399	EGR2	Early growth response 2	100	60
BG326045	BHLHB2	Basic helix-loop-helix domain containing	6.6	2.7
M60721	HLX1	H2.0-like homeo box 1	4.0	2.1
AL120562	KLF7	Kruppel-like factor 7	3.3	1.5
BF436898	ETV6	Ets variant gene 6 (TEL oncogene)	3.1	1.7
NM_005251	FOXC2	MFH-1, mesenchyme forkhead 1	2.9	1.4
NM_012099	CD3EAP	CD3-epsilon-associated protein	2.7	1.6
AF247704	NKX3-1	NK3 transcription factor related	2.5	1.3
**Other functions**				
AW138350	ICHTHYIN	Ichthyin	33.1	17.8
AW004016	ST6GAL2	Beta-galactosamide a-2,6-sialyltransferase 2	12.6	7.5
AF153330	SLC19A2	Thiamine transporter	5.6	2.6
AK026466	CYFIP2	Cytoplasmic FMR1 interacting protein 2	5.0	2.5
AK026905	MICAL2	Microtubule associated monoxygenase	3.6	1.5
NM_000427	LOR	Loricrin	3.2	1.3
AF043472	KCNS3	Potassium voltage-gated channel	2.7	1.4
NM_020127	TUFT1	Tuftelin	2.7	1.6
AI628360	NPR3	Natriuretic peptide receptor C	2.2	1.2
AI091372	AXUD1	TGF-beta-induced apoptosis protein 3	2.2	1.3

Human foreskin fibroblasts were treated with medium or 5 ng/ml TGF- ß1 for 3 hours in the presence and absence of *T. cruzi* PCM.

TGF-ß1 treatment resulted in a ≥2-fold up-regulation of 298 unique genes (represented in 309 probe-sets) as compared to cells treated with medium. Genes highlighted above represent the 59 annotated, non-redundant TGF-ß-inducible genes for which expression is reduced by 1.7-fold or greater in the presence of *T. cruzi* PCM in 3 independent experiments. * Values represent the ratio of the normalized log_2_ intensities for treatment (TGF-ß or TGF-ß/PCM)/mock treated controls.

**Figure 5:**
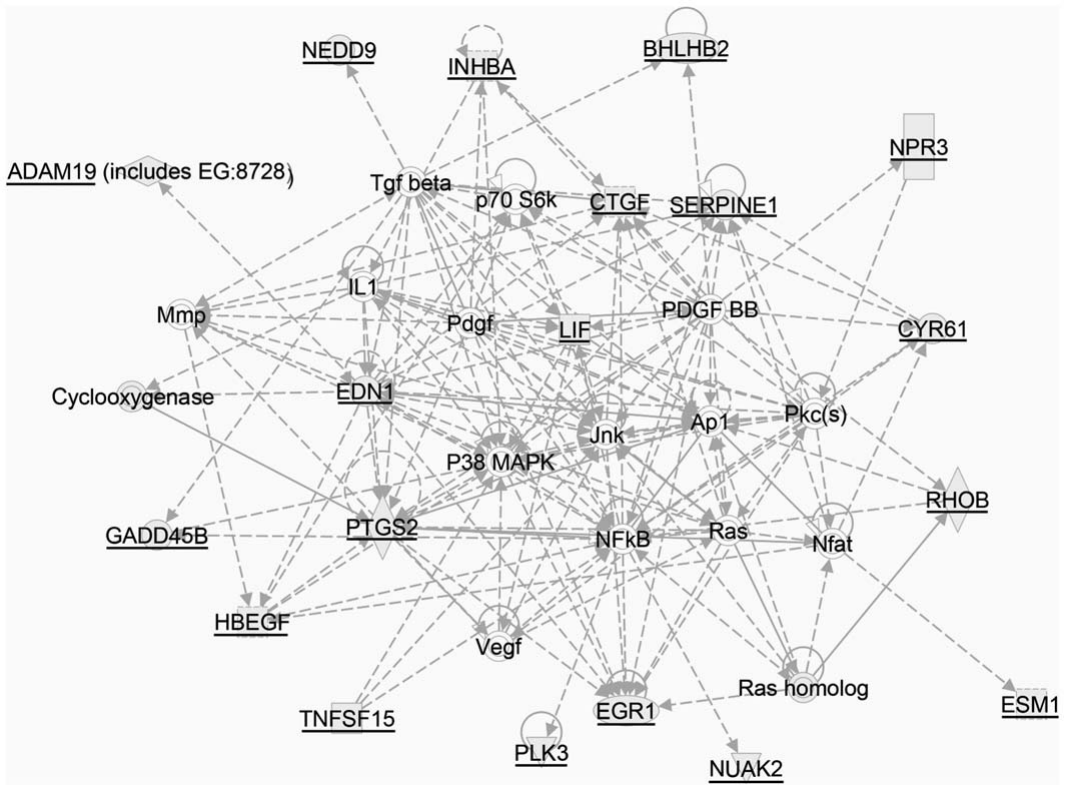
Top biological network relevant to the subset of PCM-sensitive TGF-ß-inducible genes. Biological network was generated through the use of Ingenuity Pathway Analysis software (Ingenuity® Systems, www.ingenuity.com) by uploading the group of TGF-ß-inducible genes that were highly sensitive to T. cruzi PCM. The analysis reports direct (solid lines) and indirect (dotted lines) relationships between genes as established in the literature. Thirteen (shaded symbols) of 15 genes entered into the analysis were included as focus genes in this network.

Biological network analysis conducted using the Ingenuity Pathway Analysis™ software program reveals a dense set of relationships between the subset of *T. cruzi* PCM sensitive TGF-ß-inducible genes and MAP kinases (Figure 5) where the top functions associated with this network include ‘cellular growth’, ‘cancer’ and ‘connective tissue development and function’. A comparative analysis of the top canonical pathways represented in the *T. cruzi* PCM-sensitive and PCM-insensitive TGF-ß inducible gene datasets are shown in Table S3. The top canonical pathways associated with the PCM-sensitive genes reveal 5 pathways with significant p-values (p<0.05) including ‘hepatic fibrosis/hepatic stellate cell activation’ (*CTGF, FN1, TGFb2, EDN1, CCL2, IL-6*) as the most significant pathway affected by the secreted/shed *T. cruzi* fraction. In contrast, when the PCM-insensitive group of TGF-ß-inducible genes was considered, the top functions involved ‘axonal guidance’ and ‘clathrin-mediated endocytosis signaling’ (Table S4) [43], [44], [45], [46], [47], [48], [49]. Collectively, the global expression data are supportive of a model in which infective *T. cruzi* trypomastigotes secrete or release factors that within minutes dampen MAP kinase signaling in human dermal fibroblasts. We propose that this dampening of host cell MAP kinase activity negatively impacts the activities of downstream transcription factors such as the ETS family of transcription factors known to be involved in the regulation of a number of genes involved in ECM synthesis and wound repair [50], [51] including *ctgf/ccn2* gene expression [21], [22], [51].

## Discussion

This study demonstrates that mammalian-infective forms of *Trypanosoma cruzi* release a factor (or factors) that significantly impacts host cell signaling cascades, thereby altering the expression of a subset of genes involved in cell proliferation, wound repair and inflammation. Focusing on the pro-fibrogenic cytokine, CTGF/CCN2, a multifunctional secreted protein with a central role in wound repair and fibrosis [9], we demonstrate that a shed/secreted *T. cruzi* factor inhibits both basal and agonist-induced up-regulation of *ctgf/ccn2* gene expression. In line with previous reports demonstrating a critical role for MAP kinases in regulating *ctgf/ccn2* gene expression in response to pro-fibrogenic agonists [15], [17], [18], [19], [20], our data demonstrate a role for Erk1/2, JNK and p38 in TGF-ß-mediated *ctgf/ccn2* expression in low passage primary HFF. Exposure of HFF to the shed/secreted *T. cruzi* fraction inhibits basal and agonist-dependent phosphorylation of Erk1/2, JNK and p38 and the induction of *ctgf/ccn2* expression, a downstream target of MAP kinase activation [15]. Analysis of the global transcriptional response to *T. cruzi* PCM in HFF revealed significant repression of a subset of TGF-ß-inducible genes involved in cell proliferation, wound healing and inflammatory responses, where the genes most sensitive to *T. cruzi*-mediated inhibition (*EGR1*, *HBEGF*, *PTGS2*, *LIF*, *EDN1*, *IL6*, and *CYR61)* are known to be regulated by MAP kinase-activated transcription factors such as the ETS-family members and AP-1 [43], [44], [45], [46], [47], [48], [49]. In addition, genes encoding components of the AP-1 complex, *c-jun* and *c-fos,* as well as *egr-1* were also rapidly down-regulated in *T. cruzi* PCM treated cells, suggesting the disruption of an important regulatory network centered around MAP kinase signaling, which is required for efficient expression of *ctgf/ccn2* and a host of other TGF-ß-inducible genes. Consistent with the notion that *T. cruzi* might negatively regulate MAP kinase-activated transcription factors, we demonstrated that a functional Ets-binding site (spanning −91 and −84 bp) in the upstream *ctgf/ccn2* promoter region, which is critical for TGF-ß stimulated expression of *ctgf/ccn2* [15], [21], [22], is required for *T. cruzi* PCM repression of both basal and TGF-ß-stimulated reporter expression. Thus, we propose that the observed inhibition of agonist-induced phosphorylation of the MAP kinases, Erk, p38 and JNK, would impact the activation of MAP kinase-activated transcription factors such as Ets-1. Failure to assemble Ets transcription factor(s) at the Ets binding site, possibly in conjunction with other accessory proteins such as AP-1 [21], would prevent activation of the *ctgf/ccn2* promoter in response to signals mediated by exogenous agonists such as TGF-ß [15]. Overall, our findings reinforce recent literature regarding the critical role for MAP kinase signaling in regulation of *ctgf/ccn2* expression [9], [18], [20] as well as for the role of the Ets-binding site on the *ctgf/ccn2* promoter [21], [22] in driving *ctgf/ccn2* gene expression. Given that CTGF/CCN2 is a therapeutic target for the prevention and treatment of a number of disease states in which the expression of this critical cytokine is dysregulated [10], [11], [12], [13], our finding that the mammalian-infective forms of *T. cruzi* release a factor that interferes with agonist-dependent up-regulation of CTGF/CCN2 warrants further study.

We had shown previously that infection with live *T. cruzi* trypomastigotes or parasite-conditioned medium (PCM) causes rapid dephosphorylation of Erk1/2 and sustained repression of extracellular matrix protein expression in dermal fibroblasts [7]. Focusing on the secreted/released *T. cruzi* fraction in this study, we demonstrate the broad impact of *T. cruzi* PCM on MAP kinase signaling where agonist-dependent activation of Erk1/2, p38 and JNK is thwarted, as is the expression of a subset of TGF-ß-dependent gene expression. Our findings are consistent with a recent study demonstrating that *T. cruzi* infection of primary cardiomyocytes renders infected cells refractory to TGF-ß-induced increases in fibronectin expression [52]. While these *in vitro* observations provide novel insights into the impact of infective *T. cruzi* trypomastigotes on host cell signaling and gene expression, the ultimate goal would be to measure these events and outcomes in the context of an *in vivo* infection. Given the critical role played by MAP kinases in cell proliferation, wound healing and host immunity [19], [53], [54], [55]. It is tempting to speculate that modulation of this signaling pathway early in the *T. cruzi* infection process could modulate the local host environment in favor of the parasite. If, as suggested by *in vitro* studies, ECM synthesis and host immune response pathways were to be repressed at local sites of *T. cruzi* infection, we would predict that these events would facilitate the early establishment of infection in the host. While this would be challenging to test in the context of ECM homeostasis, the concept of immune evasion early in the establishment of infection by *T. cruzi* trypomastigotes is not new [56]. There are several examples of soluble *T. cruzi* immunomodulatory molecules including the trypanosomal immunosuppressive factor (TIF) which represses T- and B-cell functions [57], [58], [59], [60] and a mucin-like protein expressed on the surface of epimastigotes and metacyclic trypomastigotes (AgC10) which inhibits LPS-mediated signaling and dendritic cell activation via MAP kinase [61]. A parasite glycoinositol-phospholipid [62] and another polypeptide antigen (Ag123) [63] have also been implicated in modulating the host immune response demonstrating that *T. cruzi* may have evolved several overlapping mechanisms for interfering with normal host immune function. The *T. cruzi* molecule(s) responsible for antagonizing TGF-ß signaling in dermal fibroblasts, that dampen fibrogenic and immune gene expression, have yet to be identified. Efforts focused on isolation of this heat-labile, trypsin-sensitive factor have revealed that it co-fractionates with the major parasite surface glycoproteins and elutes from an anion exchange column in a sharp peak with ∼200 mM NaCl (A. Mott and B. Burleigh, unpublished observations) and is likely a component of previously described small shed vesicles [64]. It will be of great interest to identify the molecular basis for the parasite-dependent repression to better assess its functional properties and to probe its potential as a novel antagonist of fibrogenic gene expression and potential immunosuppressive activity *in vitro* and *in vivo*.

Overall, this study highlights the ability of a trypomastigote-associated soluble factor to dampen basal and agonist-induced expression of a key regulator of ECM synthesis as well as genes involved in wound repair and host response to infection. As studies have clearly demonstrated, *T. cruzi* infection eventually elicits a robust inflammatory response in the host where elevated ECM synthesis and fibrosis are known to contribute to the pathophysiology of Chagas' disease (reviewed in [65]). The cellular responses to *T. cruzi* released/secreted factors studied here are immediate/early in nature and provide insights into potential processes that would be expected to occur at early time points of infection, where the parasite-mediated inhibition of host cell fibrogenic gene expression may serve a critical function in facilitating dissemination from the initial sites of infection to the circulation and peripheral organs during acute stages of infection.

## Materials and Methods

### Cell Culture and Parasite Maintenance

Human foreskin fibroblast (HFF) BJ cells were acquired from the American Tissue Culture Collection (Manassas, VA, USA) and maintained in DMEM +10% fetal bovine serum (FBS), 1% pen-strep, and 2 mM glutamine at 37°C in 5% CO_2_. Tissue culture-derived *Trypanosoma cruzi* trypomastigotes (Y strain) were generated by weekly passage in confluent monolayers of LLcMK_2_ cells in DMEM containing 2% FBS as described [5]. Trypomastigotes harvested from cell culture supernatants were washed 3 times in cold serum-free DMEM (SF-DMEM), resuspended in warm SF-DMEM at a concentration of 5×10^7^ to 1×10^8^ parasites per ml and incubated at 37°C in 5% CO_2_ for 14–18 hours. The resulting ‘parasite-conditioned medium’ (PCM) was clarified by centrifugation of parasites (1000xg) followed by passage through a 0.2 µm membrane. Prior to treatment of cells, PCM is adjusted to pH 7.4 by addition of a small volume of 1M Tris pH 8.0 and supplemented with heat-inactivated FBS (95°C, 15 minutes) to achieve a final concentration of 2%.

### Quantitative Real-Time PCR

HFF were seeded in 6-well plates and grown to 80–90% confluence for 48 hours prior to treatment of cells as indicated. Following treatment, cells were washed with cold PBS and total cellular RNA was harvested using the RNeasy RNA isolation system (Qiagen, Valencia, CA, USA) according to manufacturer's instructions. 200 ng of DNaseI-treated RNA (Invitrogen, Carlsbad, CA, USA) was transcribed to cDNA using the iScript Reverse Transcription system (BioRad, Hercules, CA, USA). Multiplex quantitative real-time PCR was carried out using an ABI 7300 instrument. The amount of experimental cDNA was normalized to GAPDH levels present in the same sample. For quantification of CTGF/CCN2 mRNA each reaction included 20 nM of each primer (forward 5′-CTGCCCTCGCGGCTTA-3′ and reverse 5′GGACCAGGCAGTTGGC TCTA-3′) and 10 nM FAM-labeled CTGF/CCN2 probe (6FAM-ACACGTTTG GCCCAGACCCAACTATG-TAMRA). Primers and probes specific for human SMAD7, SERPINE-2, CDKN2B, Cyr61, HBEGF, PTSG2 and GAPDH gene assays were obtained from Applied Biosystems Inc. (Foster City, CA, USA).

### Western blot Analysis

Relative expression of CTGF/CCN2 was determined in HFF that were serum-starved for 18 hours prior to treatment with 5 ng/ml TGF-ß for 24 hours in the presence or absence of PCM. Cells were rinsed in cold PBS, lysed directly in 2X Laemmli Buffer containing 5% ß-mercaptoethanol (BioRad, Hercules, CA, USA) and western blot analyses were performed. For MAP kinase activation, HFF were serum starved for 18 hours prior to treatment with 10 mM LPA or 5 ng/ml TGF-ß1 with or without PCM for 5 or 15 minutes. Anti-ß-actin antibodies were purchased from Sigma (St. Louis, MO, USA); anti-CTGF from Santa Cruz Biotechnology (Santa Cruz, CA, USA); anti-p44/42, anti-phospho-p44/p42, anti-p38, anti-phospho-p38, anti-SAPK/JNK and anti-phospho-SAPK/JNK from Cell Signaling Technology (Danvers, MA, USA). The ECL Plus chemiluminescence kit (Amersham Pharmacia, Arlington Heights, IL, USA) was used for detection. Blots were scanned and band intensity was quantified using the software program UN-SCAN-IT gel (Silk Scientific Corporation, Orem, UT, USA).

### Transient Transfection and Reporter Assays

HFF were seeded in 6-well plates and grown to 50% confluence for 24 hours. Cells were transfected with 1 µg of experimental plasmid DNA and 0.05 µg of control plasmid by lipofection (FuGENE 6, Roche, Indianapolis, IN, USA) for 3–4 hours in DMEM +10% FBS. Cells were washed and incubated in DMEM +10% FBS for an additional 12–14 hours prior to incubation for 6 hours with DMEM-2, PCM prepared in DMEM-2, 5 ng/ml of TGF-ß1 in the presence and absence of PCM. Promoter/reporter constructs used contained *ctgf/ccn2* promoter fragments spanning nucleotides −805 to +17 *(*−*805*), −244 to +17 (−*244*), −86 to +17 (−*86*) and a construct identical to 805 with a mutation in the Ets binding site (−*805 ETS*) corresponding to a GGAAT to TCCCG change in the consensus Ets binding motif located in the transcription enhancing factor (TEF) binding element located between −91 and −84. All constructs were kindly provided by Dr. A. Leask [21], [27]. Transfection efficiency was measured by co-transfection with pSV-ß-Galactosidase plasmid. Cells were harvested and SEAP and ß-galactosidase assays were performed using the Luminescent ß-galactosidase Reporter System or Great EscAPe SEAP Chemiluminescence Kit (Clontech, Mountain View, CA, USA) according to manufacturer's instructions.

### DNA Microarray Hybridization and Analysis

HFF were mock-treated or stimulated with TGF-ß for 3 hours, in the presence or absence of *T. cruzi* PCM, rinsed 3 times with PBS. RNA was isolated with Trizol reagent (Invitrogen, Carlsbad, CA, USA) and concentrated using an RNeasy kit (Qiagen, Valencia, CA, USA). RNA integrity was evaluated employing an Agilent 2100 Bioanalyzer (Agilent Technologies, Santa Clara, CA, USA), three independent samples for each treatment were processed and hybridized to individual HG_U133 plus 2.0 Affymetrix chips at the Harvard Medical School Biopolymers Facility. Arrays were scanned using a GeneChip® Scanner 3000 (Affymetrix, Santa Clara, CA, USA) and data files containing the unprocessed intensity values were imported into Rosetta Resolver Biosoftware 7.0. Data were pre-processed to reduce systematic errors (i.e. background subtraction and intra-array normalization). Through the application of the Affymetrix-specific error model within Rosetta Resolver [66] the data were transformed into profiles (i.e. scanned, imaged and normalized expression data from a microarray as described in http://www.rosettabio.com/tech/Data_processing_and_analysis_methods.pdf). Intensity ratios were calculated from the statistical combination of replicate profiles in order to increase the confidence in measurements and to obtain fold change values and associated p*-*values, using the control condition (cells treated with culture medium) as a baseline. Transcripts showing ≥2-fold change (p<0.01) with respect to matched controls were used to create gene lists and for analysis of biological networks using Ingenuity Pathway Analysis™ software (http://www.ingenuity.com). In accordance to the minimum information about microarray experiment (MIAME) guidelines, the complete raw and processed data files for each array are publicly available at the Gene Expression Omnibus (GEO) database repository (http://www.ncbi.nlm.nih.gov/geo) under accession number GSE16416.

## Supporting Information

Table S1 **Modulation of fibroblast gene expression by a secreted/released** ***T. cruzi*** **fraction.** Human foreskin fibroblasts (HFF) were mock-treated or incubated with cell-free *T. cruzi* parasite-conditioned medium (PCM) for 3 hours and the isolated RNA was processed and used to hybridize HG_U133 plus 2.0 Affymetrix arrays. Ratios of the normalized log_2_ intensities for PCM vs medium were used to calculate the fold-change in HFF gene expression for each array and values from 3 biological replicates were averaged. HFF genes for which the transcript abundance changes ≥2-fold in either direction are included.
(XLSX)

Table S2 **Inhibition of TGF-ß-inducible gene expression by** ***T. cruzi*** **PCM**. HFF were mock-treated or stimulated with 5 ng/ml TGF-ß1 for 3 hours in the presence or absence of *T. cruzi* PCM and cells were processed for microarray hybridization as described. Ratios of the normalized log_2_ intensities for TGF-ß vs medium and TGF-ß+PCM vs medium were used to calculate the fold-decrease in TGF-ß inducible gene expression that was observed in the presence of *T. cruzi* PCM. Only the non-redundant, annotated genes are shown with the exception of genes for which duplicates showed marked differences in the fold-change values.
(XLSX)

Table S3 **Canonical pathways enriched in the** ***T. cruzi*** **PCM-sensitive subset of TGF-ß inducible genes**. TGF-ß-inducible genes sensitive to *T. cruzi* PCM (59 genes from Table 1) were analyzed using Ingenuity Pathway Analysis software. Canonical pathways were identified from the Ingenuity Pathways Analysis library of canonical pathways that were most significant to the data set and associated with a canonical pathway in Ingenuity's Knowledge Base. The significance of the association between the data set and the canonical pathway was measured in 2 ways: 1) A ratio of the number of molecules from the data set that map to the pathway divided by the total number of molecules that map to the canonical pathway is displayed. 2) Fisher's exact test was used to calculate a p-value determining the probability that the association between the genes in the dataset and the canonical pathway is explained by chance alone.
(XLS)

Table S4 **Canonical pathways enriched in the subset of TGF-ß inducible genes that is insensitive to** ***T. cruzi*** **PCM**. TGF-ß-inducible genes that were insensitive to *T. cruzi* PCM (192 genes; Table S2) were analyzed using Ingenuity Pathway Analysis software and canonical pathways were identified from the Ingenuity Pathways Analysis library as described for Table S3.
(XLS)
